# Impact of Bariatric Surgery on Postoperative Outcomes, Complications, and Revision Rates in Total Knee Arthroplasty: A Big Data Analysis

**DOI:** 10.3390/jcm14041187

**Published:** 2025-02-11

**Authors:** David Maman, Guy Eynhoren, Lior Ben-Zvi, Yaniv Steinfeld, Yaniv Yonai, Yaron Berkovich

**Affiliations:** 1 Carmel Medical Center, Haifa 3436212, Israel; liorbenzvi@gmail.com (L.B.-Z.); yanivsteinfeld@gmail.com (Y.S.); yanivyonai@gmail.com (Y.Y.); yaron.berkovich@gmail.com (Y.B.); 2 Technion Israel Institute of Technology, Haifa 2611001, Israel; guy.eynhoren@campus.technion.ac.il

**Keywords:** total knee arthroplasty, obesity, bariatric surgery, postoperative complications, revision surgery

## Abstract

**Background:** Obesity is a major risk factor for osteoarthritis (OA) and negatively impacts both short-term and long-term outcomes following total knee arthroplasty (TKA). Bariatric surgery has been proposed as a strategy to improve surgical outcomes in obese patients; however, its effects on postoperative complications, hospital stay, healthcare costs, and revision rates remain insufficiently explored. This study investigates whether bariatric surgery before TKA is associated with improved outcomes. **Methods:** This retrospective cohort study utilized data from the Nationwide Inpatient Sample (NIS) from 2016 to 2019, including 2,519,099 TKA patients, of whom 56,291 had a history of bariatric surgery. Propensity score matching was applied to balance baseline characteristics between groups. Statistical analyses compared the length of hospital stay (LOS), total healthcare costs, postoperative complications, and revision rates. **Results:** Patients with a history of bariatric surgery exhibited significantly lower rates of sepsis, deep vein thrombosis (DVT), pulmonary embolism (PE), acute kidney injury (AKI), and ileus compared to those without. Additionally, these patients had a shorter LOS and slightly lower total hospital charges. However, the bariatric surgery group had higher rates of blood loss anemia, intraoperative fractures, and blood transfusions. Revision surgery rates were also higher in the bariatric surgery group, with mechanical loosening and prosthesis instability being more common etiologies. **Conclusions:** Bariatric surgery is associated with fewer early postoperative complications and shorter hospital stays in TKA patients, suggesting potential perioperative benefits. However, increased risks of blood loss anemia, intraoperative fractures, and revision surgery highlight the need for further research on long-term outcomes and alternative weight-loss strategies, such as pharmacologic interventions.

## 1. Introduction

Osteoarthritis (OA) is the most common form of arthritis [1], affecting approximately 528 million people worldwide in 2019 [2]. The knee is the most frequently impacted joint by OA [2]. Among treatment options, total knee arthroplasty (TKA) is widely regarded as the most definitive and effective intervention compared to non-surgical approaches [3,4]. Additionally, TKA is one of the most commonly performed and rapidly increasing surgical procedures globally, with an estimated 1.3 million procedures predicted in the near future [5,6].

Obesity, a well-established risk factor for OA, accelerates joint degeneration and is disproportionately prevalent among TKA candidates [7,8,9]. The global rise in obesity is expected to further increase the incidence of OA [10]. Obesity not only contributes to the development of OA but also negatively impacts both short-term and long-term outcomes following TKA [11,12,13]. Severely obese patients often experience worse outcomes than those at a reference weight, though the benefits of addressing obesity prior to TKA remain unclear [14,15]. While diet-induced weight loss has been associated with improved outcomes, such interventions typically result in less significant weight loss and involve patients with a lower body mass index (BMI) compared to those undergoing bariatric surgery [16].

Bariatric surgery, known for achieving sustained and significant weight loss, has been proposed as a potential strategy to improve TKA outcomes [17,18]. This procedure can result in a 15% to 30% reduction in total body weight over a decade [19], far exceeding the outcomes of non-surgical weight loss programs [20,21]. However, the impact of prior bariatric surgery on postoperative complications, healthcare costs, length of hospital stay, and revision rates following TKA remains underexplored.

To date, most studies that demonstrate improved outcomes following TKA in patients with a history of bariatric surgery have relied on smaller-scale trials and have only limitedly addressed postoperative complications. Our study aims to fill this gap by leveraging big data analytics to comprehensively evaluate TKA outcomes in patients with a history of bariatric surgery. We will explore whether bariatric surgery reduces postoperative complications, healthcare costs, hospital length of stay, and revision surgery rates. Ultimately, our goal is to enhance clinical, functional, and quality-of-life outcomes for severely obese patients undergoing TKA.

This study leverages big data analytics from the NIS database (2016–2019) to evaluate the impact of bariatric surgery on TKA outcomes, addressing gaps in knowledge regarding postoperative complications, healthcare costs, LOS, and revision rates.

### Research Questions

Does a history of bariatric surgery improve postoperative outcomes, including complications, healthcare costs, hospital length of stay, and revision rates, in severely obese patients undergoing total knee arthroplasty compared to those without prior bariatric surgery?

## 2. Methods

### 2.1. Dataset Acquisition

This study utilized a comprehensive dataset extracted from the Nationwide Inpatient Sample (NIS), the largest publicly available all-payer inpatient care database in the United States. The dataset included a total of 2,519,099 patients; of them, 56,291 patients underwent TKA with a history of bariatric surgery.

### 2.2. Study Period and Data Source

The study period spanned from 1 January 2016 to 31 December 2019, representing the latest available information within the NIS system at the time of this study. The NIS, a core component of the Healthcare Cost and Utilization Project (HCUP), captures 20% of inpatient stays from HCUP-associated hospitals, amounting to approximately seven million unweighted enrollments annually.

Strengths of NIS:

- Large sample size, minimizing selection bias;
- Inclusion of diverse hospital types (academic, non-academic, rural, urban);
- Reliable ICD-10 coding for procedural and diagnostic data.

Limitations of NIS:

- No long-term follow-up beyond the hospital stay;
- Reliance on administrative coding, which may introduce errors;
- Lack of granular clinical details, such as specific surgical techniques.

### 2.3. Patient Identification and Exclusions

Patients undergoing TKA were identified based on ICD-10 coding related to these procedures. Exclusions comprised patients with non-elective admissions or those who underwent surgery before admission.

### 2.4. Statistical Analyses and Propensity Score Matching

Statistical analyses, including crosstabs and independent sample *t*-tests, were conducted using SPSS 26 and MATLAB 2024 to compare patients with and without a history of bariatric surgery. A significance level of *p* < 0.05 was applied. To address potential variations and selection bias, a propensity score-matched analysis was performed using MATLAB. The refined dataset included 56,291 cases for each group with comparable characteristics undergoing TKA with or without a history of bariatric surgery. 1:1 nearest-neighbor propensity score matching was applied with a caliper width of 0.01. Matching variables included: demographics (age, gender, payer type), hospital characteristics (size, teaching status, urban/rural location), and comorbidities (hypertension, diabetes, dyslipidemia, chronic lung disease), and standardized mean differences (SMD) were calculated to confirm statistical balance between groups post-matching.

### 2.5. Comorbidity and Outcome Identification

Comorbidities were identified through a review of patient-specific ICD-10 codes to discern trends and derive key statistical insights. TKA procedures were delineated using specific ICD-10-PCS codes. Clinical outcomes, including in-hospital mortality, length of stay, complications, and overall hospitalization costs, were analyzed using established methodologies based on specific ICD-10 codes. The complications measured included blood transfusion, sepsis, deep vein thrombosis (DVT), pulmonary embolism (PE), acute kidney injury (AKI), pneumonia, ileus, urinary tract infection (UTI), blood loss anemia, and intraoperative fracture.

### 2.6. Revision Surgery Analysis

A detailed analysis was conducted on patients who underwent revision surgery. The dataset included 216,380 patients without a history of bariatric surgery and 6865 patients with a history of bariatric surgery. The parameters analyzed included total numbers, age at revision, total charges, length of stay, and etiology for revision.

### 2.7. Ethical Considerations

The study received exempt status from the institutional review board, with a waiver for informed consent due to the use of de-identified data from the NIS database.

## 3. Results

**Figure 1:**
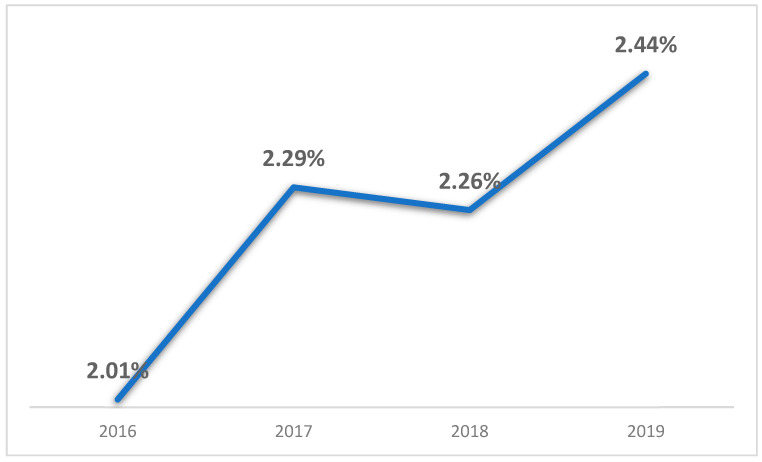
Annual Percentage of patients with history of Bariatric Surgery from all TKA Procedures (2016–2019).

There has been a notable trend in the increasing percentage of TKA patients with a history of bariatric surgery. As shown in Figure 1, the data indicates a significant upward trend in the proportion of TKA patients with a history of bariatric surgery, with a *p*-value of 0.001.

### 3.1. Comparative Analysis of TKA Patients with and Without a History of Bariatric Surgery:

Table 1 provides a comparative analysis of 2,457,804 patients undergoing TKA without a history of bariatric surgery versus 56,291 patients with a history of bariatric surgery. It outlines the distribution of surgeries, demographic details, and primary expected payers for both groups. The average age of patients undergoing TKA without a history of bariatric surgery is significantly higher at 66.9 years compared to 60.8 years for those with a history of bariatric surgery (*p* < 0.001). The proportion of female patients is significantly higher in the group with a history of bariatric surgery, at 81.7%, compared to 61.1% in the group without (*p* < 0.001).

Primary expected payer categories reveal significant differences: 57.4% of TKA patients without a history of bariatric surgery are covered by Medicare, compared to only 43.7% of those with a history of bariatric surgery (*p* < 0.001). Conversely, a larger proportion of patients with a history of bariatric surgery are covered by private insurance, including HMO, at 46.5%, compared to 34.8% for those without (*p* < 0.001).

### 3.2. Comparative Analysis of Comorbidities in TKA Patients with and Without a History of Bariatric Surgery

Table 2 provides a comparative analysis of comorbidities in TKA patients without a history of bariatric surgery. Patients with a history of bariatric surgery show significantly higher rates of obstructive sleep apnea (23.2% vs. 13%, *p* < 0.001), chronic anemia (8.4% vs. 5.7%, *p* < 0.001), osteoporosis (5.4% vs. 3.9%, *p* < 0.001), and fibromyalgia (6.4% vs. 2.6%, *p* < 0.001). They also exhibit higher prevalence of liver disease (1.7% vs. 1.2%, *p* < 0.001), disorders of the thyroid (23% vs. 17.8%, *p* < 0.001), and obesity (56.6% vs. 30.4%, *p* < 0.001).

Patients without a history of bariatric surgery have higher rates of hypertension (59.6% vs. 56.9%, *p* < 0.001), dyslipidemia (46.9% vs. 34%, *p* < 0.001), chronic kidney disease (6.9% vs. 5.9%, *p* < 0.001), and diabetes mellitus (21.6% vs. 24.4%, *p* < 0.001). Additionally, this group has higher instances of Alzheimer’s disease (0.2% vs. 0.1%, *p* = 0.698), dementia (0.5% vs. 0.2%, *p* < 0.001), and neoplasms (1% vs. 0.6%, *p* < 0.001).

### 3.3. Propensity Score-Matched Analysis of Comorbidities in TKA Patients with and Without a History of Bariatric Surgery

To mitigate potential selection bias and account for baseline differences in comorbidities, we applied propensity score matching to create statistically comparable groups. This method enhances the validity of observational studies by pairing individuals with similar probabilities of belonging to either group, thereby improving comparability. By reducing the influence of confounding variables, propensity score matching approximates the conditions of a randomized study, strengthening the reliability of our findings.

Table 3 shows a comprehensive comparison of comorbidities between patients undergoing total knee arthroplasty (TKA) without a history of bariatric surgery and those with a history of bariatric surgery after propensity score matching analysis. This analysis includes 56,291 patients in each group, ensuring a balanced comparison. The results indicate no statistically significant differences between the groups across a range of demographic and comorbidity parameters, demonstrating the effectiveness of the propensity score matching.

### 3.4. Comparison of Hospitalization Outcomes in Propensity Score-Matched TKA Patients

Hospitalization outcomes were also examined in the propensity score-matched cohorts of TKA patients with and without a history of bariatric surgery. Table 4 presents the mean length of stay and total charges for both cohorts. The findings highlight significant differences in these outcomes between the two groups, with TKA patients with a history of bariatric surgery showing shorter average hospital stays and lower total charges compared to those without a history of bariatric surgery.

### 3.5. Comparison of Postoperative Complications in Propensity Score-Matched TKA Patients

A detailed analysis of postoperative complications was conducted in the propensity score-matched cohorts of TKA patients with and without a history of bariatric surgery.

Table 5 presents the incidence rates of several key complications. Patients with a history of bariatric surgery show a higher incidence of blood transfusion (2.2% vs. 1.8%, *p* < 0.001) and blood loss anemia (17% vs. 14.2%, *p* < 0.001). The rates of sepsis (0.1% vs. 0.2%, *p* < 0.001), DVT (0% vs. 0.3%, *p* < 0.001), pulmonary embolism (0.2% vs. 0.3%, *p* < 0.001), and AKI (1.2% vs. 1.6%, *p* < 0.001) are lower in the bariatric surgery group compared to the non-bariatric surgery group. Patients with a history of bariatric surgery have a lower incidence of ileus (0% vs. 0.2%, *p* < 0.001) and UTI (0.9% vs. 1.2%, *p* < 0.001). Intraoperative fracture rates are slightly higher in the bariatric surgery group (0.3% vs. 0.2%, *p* < 0.001).

### 3.6. Comparison of Revision Surgery Outcomes in TKA Patients with and Without a History of Bariatric Surgery

A separate analysis was conducted on patients who underwent revision surgery following TKA. This group includes 216,380 patients without a history of bariatric surgery and 6865 patients with a history of bariatric surgery. Notably, the percentage of patients with a history of bariatric surgery is larger in this revision surgery cohort (3.1%) compared to the initial surgery cohort (2.3%), suggesting higher rates of revision in this group.

Table 6 presents the outcomes and etiology for revision surgery in both cohorts. Significant differences are observed in several parameters, including the age at revision and length of stay. Patients with a history of bariatric surgery tend to have revisions at a younger age (60.9 years vs. 65.5 years, *p* < 0.001) and have a longer length of stay (3.2 days vs. 3 days, *p* = 0.002).

The etiology for revision surgery also shows significant differences. Mechanical loosening is more common in patients with a history of bariatric surgery (24.7% vs. 22.9%), as is instability of the prosthesis (16.2% vs. 11.9%). Infection rates are slightly higher in the bariatric group (22.5% vs. 22.2%).

## 4. Discussion

### 4.1. Main Findings

The primary finding of this study is that patients with a history of bariatric surgery who underwent TKA experienced significantly reduced early postoperative complications and a slightly shorter length of hospital stay compared to those without a history of bariatric surgery. This improvement in outcomes is notable despite the higher prevalence of certain comorbidities in the bariatric surgery group, such as obstructive sleep apnea, chronic anemia, osteoporosis, fibromyalgia, liver disease, thyroid disorders, and obesity.

### 4.2. Increasing Prevalence of Bariatric Surgery in TKA Patients

As the prevalence of bariatric surgery among TKA patients continues to rise (Figure 1), these findings may have increasing clinical relevance. Further research is required to confirm and strengthen the observed association between bariatric surgery history and improved postoperative outcomes in TKA patients [17,18,22,23,24]. Our data also support the conclusion that patients with a history of bariatric surgery are not worse candidates for TKA compared to overweight patients without bariatric surgery [11,12,13].

### 4.3. Reduced Postoperative Complications

Our study demonstrates a reduced incidence of complications such as sepsis, DVT, PE, AKI, ileus, and UTI in the bariatric surgery group. These results highlight the potential benefits of undergoing bariatric surgery prior to TKA in reducing early postoperative complications. Additionally, the shorter LOS observed in the bariatric surgery group (2.36 days) compared to the non-bariatric group (2.58 days) is clinically significant, suggesting that a history of bariatric surgery may promote faster recovery due to fewer complications [25,26]. This reduction in hospital stay also has implications for decreasing healthcare costs and promoting more efficient resource utilization.

### 4.4. Increased Risk of Blood-Related Complications

However, despite these advantages, certain postoperative complications were more frequent in the bariatric surgery group, including blood loss anemia, intraoperative fractures, and an increased need for blood transfusions. The higher incidence of anemia-related complications is likely linked to bariatric surgery-induced malabsorption of nutrients, particularly iron, which can result in iron deficiency anemia [27,28,29]. The increased rate of intraoperative fractures may be related to long-term effects of bariatric surgery, such as vitamin D and calcium malabsorption, which weaken bone health [30,31,32,33].

### 4.5. Revision Surgery and Long-Term Outcomes

Patients with a prior history of bariatric surgery exhibited higher rates of revision surgery, a finding consistent with previous research [22,34]. Manli Yan et al. [34] reported that short-term revision rates remained stable, while long-term revision rates increased. In our study, the primary etiological differences between patients with and without a history of bariatric surgery were higher rates of mechanical loosening (24.7% vs. 22.9%) and prosthesis instability (16.2% vs. 11.9%) in the bariatric group. These findings may be attributed to long-term metabolic complications following bariatric surgery, including vitamin D and calcium deficiencies, which can impair bone mineralization and periprosthetic stability over time [30,31,32,33].

Additionally, weight fluctuations and potential weight regain following bariatric surgery [35] may place increased biomechanical stress on the prosthesis, contributing to implant failure and revision. Another important consideration is the potential impact of bariatric surgery on bone cement degradation and fixation quality. Studies have suggested that altered bone metabolism, lower bone mineral density, and nutritional deficiencies in post-bariatric surgery patients may lead to accelerated cement wear, impaired cement-bone integration, and higher rates of aseptic loosening [36,37,38]. These mechanisms could explain the increased rates of mechanical loosening and implant instability observed in our study.

Despite these findings, the true long-term impact of bariatric surgery on TKA revision rates remains uncertain, as our study is limited to in-hospital outcomes. Further prospective, longitudinal studies are necessary to fully assess the long-term durability of prostheses in post-bariatric surgery patients and determine whether alternative weight-loss strategies, such as pharmacologic interventions, may offer comparable benefits with fewer long-term orthopedic complications. Additionally, future research should investigate the role of cemented vs. uncemented fixation in post-bariatric surgery patients to determine whether specific fixation methods influence implant survival rates.

### 4.6. Short-Term Benefits vs. Long-Term Risks

Our study highlights the dual effect of bariatric surgery before TKA. In the short term, patients with a history of bariatric surgery had fewer perioperative complications, lower rates of sepsis (0.1% vs. 0.2%), DVT (0% vs. 0.3%), PE (0.2% vs. 0.3%), AKI (1.2% vs. 1.6%), and shorter hospital stays. These findings align with prior research suggesting that weight reduction reduces systemic inflammation, enhances perioperative hemodynamics, and lowers the overall surgical risk.

However, in the long term, our study found increased risks for revision surgery (3.1% vs. 2.3%), likely due to bone density loss, altered biomechanics, and potential weight regain. This is further supported by higher rates of mechanical loosening (24.7% vs. 22.9%) and prosthesis instability (16.2% vs. 11.9%) in the bariatric group. These findings suggest that while bariatric surgery provides short-term advantages, it may predispose patients to long-term implant-related complications that require further investigation.

### 4.7. Limitations

First, the NIS dataset lacks long-term follow-up beyond hospitalization, making it impossible to track complications occurring months or years after surgery. Second, reliance on ICD-10 coding introduces the possibility of misclassification bias, particularly in identifying complications and revision surgeries [35,36,37,38]. Third, BMI and weight at the time of surgery are not available in the dataset, limiting our ability to assess the direct impact of preoperative body weight, weight loss maintenance, or weight regain on TKA outcomes. Given the strong association between BMI and joint biomechanics, future research should incorporate longitudinal weight data to better understand these relationships. Finally, our study does not differentiate between types of bariatric surgery (e.g., gastric bypass vs. sleeve gastrectomy), which may impact long-term orthopedic outcomes differently.

The strengths of this study include the use of a large, nationally representative dataset and the application of rigorous statistical methods to ensure robust and reliable results. The findings contribute to the growing body of evidence supporting the benefits of bariatric surgery history before TKA in improving patient outcomes and reducing healthcare costs.

### 4.8. Future Directions

Further research is necessary to address the long-term effects of bariatric surgery on TKA outcomes. Future studies should:

○ Include longitudinal follow-up data to evaluate implant survival rates in post-bariatric patients;
○ Differentiate between various bariatric surgery types to assess whether specific procedures influence TKA outcomes differently;
○ Compare bariatric surgery with pharmacological weight-loss strategies, such as GLP-1 receptor agonists (e.g., Ozempic, Wegovy), to determine if similar benefits can be achieved without the risks of malabsorption and osteoporosis.

## 5. Conclusions

Bariatric surgery before TKA is associated with shorter hospital stays and fewer early complications, including lower rates of sepsis, PE, AKI, and DVT. However, our study also highlights higher risks of blood loss anemia, intraoperative fractures, and increased revision surgery rates, primarily due to mechanical loosening and prosthesis instability. These findings suggest that while preoperative weight loss through bariatric surgery may improve short-term recovery, careful patient selection is necessary to mitigate long-term risks.

Given the potential drawbacks of bariatric surgery, future studies should investigate alternative weight-loss strategies, such as GLP-1 agonists, to determine whether similar perioperative benefits can be achieved without increased long-term revision rates.

## Figures and Tables

**Table 1:** Demographic and Payer Characteristics of TKA Patients with and without a history of bariatric surgery.

Parameter	No History of Bariatric Surgery	History of Bariatric Surgery	Significance
Total Surgeries	2,457,804	56,291	-
Average Age (y)	66.9	60.8	*p* < 0.001
Female (%)	61.1	81.7	*p* < 0.001
Primary expected payer—Medicare (%)	57.4%	43.7%	*p* < 0.001
Primary expected payer—Medicaid (%)	4.2%	6.0%	
Primary expected payer—private including HMO (%)	34.8%	46.5%	
Primary expected payer—self-pay (%)	0.5%	0.5%	
Primary expected payer—no charge (%)	0.0%	0.0%	
Primary expected payer—other (%)	3.1%	3.2%	

**Table 2:** Prevalence of comorbidities in TKA Patients with and without a History of Bariatric Surgery.

Parameter	No History of Bariatric Surgery	History of Bariatric Surgery	Significance
Hypertension (%)	59.6	56.9	*p* < 0.001
Dyslipidemia (%)	46.9	34	*p* < 0.001
Obstructive Sleep Apnea (%)	13	23.2	*p* < 0.001
Chronic Anemia (%)	5.7	8.4	*p* < 0.001
Alcohol Abuse (%)	0.9	1.1	*p* < 0.001
Osteoporosis (%)	3.9	5.4	*p* < 0.001
Parkinson Disease (%)	0.6	0.3	*p* < 0.001
Alzheimer Disease (%)	0.2	0.1	*p* = 0.698
Chronic Kidney Disease (%)	6.9	5.9	*p* < 0.001
Congestive Heart Failure (%)	1.2	1.6	*p* < 0.001
Chronic Lung Disease (%)	5.9	5.6	*p* < 0.001
Diabetes Mellitus (%)	21.6	24.4	*p* < 0.001
IBD (%)	0.5	0.4	*p* = 0.009
Liver Disease (%)	1.2	1.7	*p* < 0.001
Obesity (%)	30.4	56.6	*p* < 0.001
Fibromyalgia (%)	2.6	6.4	*p* < 0.001
Disorders of Thyroid (%)	17.8	23	*p* < 0.001
History of Myocardial Infarction (%)	3.2	2.8	*p* < 0.001
Peripheral Vascular Disease (%)	1.4	1.5	*p* = 0.276
History of Cerebrovascular Accident (%)	4	4.2	*p* = 0.028
Dementia (%)	0.5	0.2	*p* < 0.001
Neoplasms (%)	1	0.6	*p* < 0.001
Neoplasms of Lymphoid and Hematopoietic Tissue (%)	0.4	0.3	*p* < 0.001

**Table 3:** Comparison of demographic and clinical data in propensity score-matched cohorts of TKA patients with and without a history of bariatric surgery.

Parameter	No History of Bariatric Surgery	History of Bariatric Surgery	Significance
Total Surgeries	56,291	56,291	-
Average Age (y)	60.8	60.8	0.95
Female (%)	81.8	82	*p* = 0.27
Primary expected payer—Medicare (%)	43.7	43.7	*p* = 0.58
Primary expected payer—Medicaid (%)	6.1	6.2	
Primary expected payer—private including HMO (%)	46.5	46.5	
Primary expected payer—self-pay (%)	0.5	0.5	
Primary expected payer—no charge (%)	0	0	
Primary expected payer—other (%)	3.2	3.2	
Hypertension (%)	57.2	56.9	*p* = 0.29
Dyslipidemia (%)	33.8	34	*p* = 0.47
Obstructive Sleep Apnea (%)	23	23.2	*p* = 0.38
Chronic Anemia (%)	8.2	8.4	*p* = 0.15
Alcohol Abuse (%)	1.1	1.1	*p* = 0.43
Osteoporosis (%)	5.3	5.4	*p* = 0.28
Parkinson Disease (%)	0.2	0.3	*p* = 0.08
Alzheimer Disease (%)	0.1	0.1	*p* = 0.14
Chronic Kidney Disease (%)	5.9	5.9	*p* = 0.66
Congestive Heart Failure (%)	1.5	1.6	*p* = 0.09
Chronic Lung Disease (%)	5.5	5.6	0.9
Diabetes Mellitus (%)	24.3	24.4	*p* = 0.60
IBD (%)	0.5	0.4	*p* = 0.05
Liver Disease (%)	0.9	1.7	*p* = 0.000
Obesity (%)	56.3	56.6	*p* = 0.08
Fibromyalgia (%)	6.4	6.4	*p* = 0.82
History of Myocardial Infarction (%)	2.6	2.8	*p* = 0.12
Peripheral Vascular Disease (%)	1.5	1.5	*p* = 0.75
History of Cerebrovascular Accident (%)	4.3	4.2	*p* = 0.54
Neoplasms (%)	0.5	0.6	*p* = 0.08
Neoplasms of Lymphoid and Hematopoietic Tissue (%)	0.2	0.3	*p* = 0.06

**Table 4:** Comparison of hospitalization outcomes in propensity score-matched cohorts of TKA Patients with and without a history of bariatric surgery.

	No History of Bariatric Surgery	History of Bariatric Surgery	Significance
Length of stay mean in days	2.58 (Std. deviation 1.5)	2.36 (Std. deviation 1.8)	*p* < 0.001
Total charges mean in $	62,187 (Std. deviation 36,294)	61,379 (Std. deviation 37,430)	*p* < 0.001

**Table 5:** Postoperative outcomes in propensity score-matched cohorts of TKA Patients with and without a history of bariatric surgery.

Parameter	No History of Bariatric Surgery	History of Bariatric Surgery	Significance
Blood Transfusion (%)	1.8	2.2	*p* < 0.001
Sepsis (%)	0.2	0.1	*p* < 0.001
DVT (%)	0.3	0	*p* < 0.001
Pulmonary Embolism (%)	0.3	0.2	*p* < 0.001
AKI (%)	1.6	1.2	*p* < 0.001
Pneumonia (%)	0.2	0.2	*p* = 0.739
Ileus (%)	0.2	0	*p* < 0.001
UTI (%)	1.2	0.9	*p* < 0.001
Blood Loss Anemia (%)	14.2	17	*p* < 0.001
Intraoperative Fracture (%)	0.2	0.3	*p* < 0.001

**Table 6:** Outcomes and Etiology of Revision Surgery in TKA patients with and without a history of bariatric surgery.

Revision Surgery	No History of Bariatric Surgery	History of Bariatric Surgery	
Total numbers	216,380	6865	-
%	96.9	3.1	-
Age at Revision (Years)	65.5	60.9	*p* < 0.001
Total charges ($)	97,673	102,316	*p* = 0.238
Length of stay (Days)	3	3.2	*p* = 0.002
Etiolgy for Revision			
Infection	22.2%	22.5%	*p* < 0.001
Mechanical Loosening	22.9%	24.7%	
Pain	7.4%	6.8%	
Instability of Prosthesis	11.9%	16.2%	
Wear of Articular Surface	2.1%	1.7%	
Periprosthetic Fracture	0.9%	0.7%	
Fibrosis due to Prosthetic	0.9%	0.7%	
Broken Prosthesis	1.1%	1.1%	
Surgical Wound complication	0.4%	0.2%	
Other Mechanical Complication	9.5%	8.5%	
Other/Unspecified	20.7%	16.8%	

## Data Availability

The original contributions presented in the study are included in the article; further inquiries can be directed to the corresponding author.

## Abbreviations

AKI	Acute Kidney Injury
BMI	Body Mass Index
DVT	Deep Vein Thrombosis
GLP-1	Glucagon-Like Peptide-1
HCUP	Healthcare Cost and Utilization Project
HMO	Health Maintenance Organization
IBD	Inflammatory Bowel Disease
ICD-10	International Classification of Diseases, 10th Revision
ICD-10-PCS	International Classification of Diseases, 10th Revision, Procedure Coding System
LOS	Length of Stay
NIS	Nationwide Inpatient Sample
OA	Osteoarthritis
PE	Pulmonary Embolism
TKA	Total Knee Arthroplasty
UTI	Urinary Tract Infection

## References

[1] Hunter D.J., Bierma-Zeinstra S. (2019). Osteoarthritis. Lancet.

[2] https://www.who.int/news-room/fact-sheets/detail/osteoarthritis.

[3] Gress K., Charipova K., An D., Hasoon J., Kaye A.D., Paladini A., Varrassi G., Viswanath O., Abd-Elsayed A., Urits I. (2020). Treatment recommendations for chronic knee osteoarthritis. Best Prac. Res. Clin. Anaesthesiol..

[4] Skou S.T., Roos E.M., Laursen M.B., Rathleff M.S., Arendt-Nielsen L., Simonsen O., Rasmussen S. (2015). A Randomized, Controlled Trial of Total Knee Replacement. N. Engl. J. Med..

[5] Gao J., Xing D., Dong S., Lin J. (2020). The primary total knee arthroplasty: A global analysis. J. Orthop. Surg. Res..

[6] Sloan M., Premkumar A., Sheth N.P. (2018). Projected Volume of Primary Total Joint Arthroplasty in the U.S., 2014 to 2030. J. Bone Jt. Surg..

[7] Park D., Park Y.-M., Ko S.-H., Hyun K.-S., Choi Y.-H., Min D.-U., Han K., Koh H.-S. (2023). Association of general and central obesity, and their changes with risk of knee osteoarthritis: A nationwide population-based cohort study. Sci. Rep..

[8] Shumnalieva R., Kotov G., Monov S. (2023). Obesity-Related Knee Osteoarthritis—Current Concepts. Life.

[9] Bourne R., Mukhi S., Zhu N., Keresteci M., Marin M. (2007). Role of Obesity on the Risk for Total Hip or Knee Arthroplasty. Clin. Orthop. Relat. Res..

[10] Haththotuwa R.N., Wijeyaratne C.N., Senarath U., Mahmood T.A., Arulkumaran S., Chervenak F.A. (2020). Chapter 1—Worldwide epidemic of obesity. Obesity and Obstetrics.

[11] Kerkhoffs G.M., Servien E., Dunn W., Dahm D., Bramer J.A., Haverkamp D. (2012). The Influence of Obesity on the Complication Rate and Outcome of Total Knee Arthroplasty: A meta-analysis and systematic literature review. J. Bone Jt. Surg..

[12] Deakin A.H., Iyayi-Igbinovia A., Love G.J. (2017). A comparison of outcomes in morbidly obese, obese and non-obese patients undergoing primary total knee and total hip arthroplasty. Surg..

[13] Hakim J., Volpin G., Amashah M., Alkeesh F., Khamaisy S., Cohen M., Ownallah J. (2019). Long-term outcome of total knee arthroplasty in patients with morbid obesity. Int. Orthop..

[14] Lui M., Jones C.A., Westby M.D. (2015). Effect of non-surgical, non-pharmacological weight loss interventions in patients who are obese prior to hip and knee arthroplasty surgery: A rapid review. Syst. Rev..

[15] Godziuk K., Prado C.M., Beaupre L., Jones C.A., Werle J.R., Forhan M. (2020). A critical review of weight loss recommendations before total knee arthroplasty. Jt. Bone Spine.

[16] Mishra A.K., Vaish A., Vaishya R. (2022). Effect of Body Mass Index on the outcomes of primary Total Knee Arthroplasty up to one year—A prospective study. J. Clin. Orthop. Trauma.

[17] Dowsey M.M., Brown W.A., Cochrane A., Burton P.R., Liew D., Choong P.F. (2022). Effect of Bariatric Surgery on Risk of Complications After Total Knee Arthroplasty: A Randomized Clinical Trial. JAMA Netw. Open.

[18] Stambough J.B. (2023). In Patients with Knee OA and Severe Obesity, Bariatric Surgery and Weight Loss Before TKA Reduced Complications Versus TKA Alone. J. Bone Jt. Surg..

[19] van Rijswijk A.-S., van Olst N., Schats W., van der Peet D.L., van de Laar A.W. (2021). What Is Weight Loss After Bariatric Surgery Expressed in Percentage Total Weight Loss (%TWL)? A Systematic Review. Obes. Surg..

[20] Aaseth J., Ellefsen S., Alehagen U., Sundfør T.M., Alexander J. (2021). Diets and drugs for weight loss and health in obesity—An update. Biomed. Pharmacother..

[21] Gao X., Hua X., Wang X., Xu W., Zhang Y., Shi C., Gu M. (2022). Efficacy and safety of semaglutide on weight loss in obese or overweight patients without diabetes: A systematic review and meta-analysis of randomized controlled trials. Front. Pharmacol..

[22] Ryan S.P., Couch C.G., Duong S.Q., Taunton M.J., Lewallen D.G., Berry D.J., Abdel M.P. (2022). Does Bariatric Surgery Prior to Primary Total Knee Arthroplasty Improve Outcomes?. J. Arthroplast..

[23] Wang Y., Deng Z., Meng J., Dai Q., Chen T., Bao N. (2019). Impact of Bariatric Surgery on Inpatient Complication, Cost, and Length of Stay Following Total Hip or Knee Arthroplasty. J. Arthroplast..

[24] Sattari S.A., Sattari A.R., Salib C.G., Salem H.S., Hameed D., Dubin J., Mont M.A. (2024). Total Knee Arthroplasty With or Without Prior Bariatric Surgery: A Systematic Review and Meta-Analysis. J. Arthroplast..

[25] Khan N.A., Quan H., Bugar J.M., Lemaire J.B., Brant R., Ghali W.A. (2006). Association of Postoperative Complications with Hospital Costs and Length of Stay in a Tertiary Care Center. J. Gen. Intern. Med..

[26] Chona D., Lakomkin N., Bulka C., Mousavi I., Kothari P., Dodd A.C., Shen M.S., Obremskey W.T., Sethi M.K. (2017). Predicting the post-operative length of stay for the orthopaedic trauma patient. Int. Orthop..

[27] Bjørklund G., Peana M., Pivina L., Dosa A., Aaseth J., Semenova Y., Chirumbolo S., Medici S., Dadar M., Costea D.-O. (2021). Iron Deficiency in Obesity and after Bariatric Surgery. Biomolecules.

[28] Gowanlock Z., Lezhanska A., Conroy M., Crowther M., Tiboni M., Mbuagbaw L., Siegal D.M. (2020). Iron deficiency following bariatric surgery: A retrospective cohort study. Blood Adv..

[29] Bailly L., Schiavo L., Sebastianelli L., Fabre R., Pradier C., Iannelli A. (2018). Anemia and Bariatric Surgery: Results of a National French Survey on Administrative Data of 306,298 Consecutive Patients Between 2008 and 2016. Obes. Surg..

[30] Ben-Porat T., Elazary R., Sherf-Dagan S., Goldenshluger A., Brodie R., Mintz Y., Weiss R. (2018). Bone Health following Bariatric Surgery: Implications for Management Strategies to Attenuate Bone Loss. Adv. Nutr. Int. Rev. J..

[31] Stein E.M., Silverberg S.J. (2014). Bone loss after bariatric surgery: Causes, consequences, and management. Lancet Diabetes Endocrinol..

[32] Fashandi A.Z., Mehaffey J.H., Hawkins R.B., Schirmer B., Hallowell P.T. (2018). Bariatric surgery increases risk of bone fracture. Surg. Endosc..

[33] Uebelhart B. (2016). Effects of bariatric surgery on bone. Jt. Bone Spine.

[34] Yan M., Zheng G., Long Z., Pan Q., Wang X., Li Y., Lei C. (2022). Does bariatric surgery really benefit patients before total knee arthroplasty? A systematic review and meta-analysis. Int. J. Surg..

[35] Maman D., Laver L., Becker R., Mahamid A., Berkovich Y. (2024). Robotic-assisted total knee arthroplasty reduces postoperative complications and length of stay without increased cost compared to navigation-guided techniques: A national analysis. Knee Surg. Sports Traumatol. Arthrosc..

[36] Maman D., Laver L., Becker R., Takrori L.A., Mahamid A., Finkel B., Gan-Or H., Yonai Y., Berkovich Y. (2024). Trends and epidemiology in robotic-assisted total knee arthroplasty: Reduced complications and shorter hospital stays. Knee Surg. Sports Traumatol. Arthrosc..

[37] Maman D., Mahamid A., Yonai Y., Berkovich Y. (2024). Comparing Complication Rates, Costs, and Length of Stay between Unicompartmental and Total Knee Arthroplasty: Insights from a Big Data Analysis Using the National Inpatient Sample Dataset. J. Clin. Med..

[38] Velapati S.R., Shah M., Kuchkuntla A.R., Abu-Dayyeh B., Grothe K., Hurt R.T., Mundi M.S. (2018). Weight Regain After Bariatric Surgery: Prevalence, Etiology, and Treatment. Curr. Nutr. Rep..

